# Factors Influencing Medical Students’ Decision in Choosing a Surgical Specialty

**DOI:** 10.7759/cureus.70416

**Published:** 2024-09-28

**Authors:** Jubran J Al-Faifi, Somiah A Alsarar, Rawan A Bayamin, Raghad A Alkhaldi, Hadeel S Hawsawi, Aroob M Alromih, Raghad S Alnajdi

**Affiliations:** 1 Surgery, College of Medicine, Imam Mohammad Ibn Saud Islamic University, Riyadh, SAU; 2 Medicine and Surgery, College of Medicine, Imam Mohammad Ibn Saud Islamic University, Riyadh, SAU

**Keywords:** discourage, interest, intern, medical student, motivation, surgical specialty

## Abstract

Background: The decision to pursue a career in surgery among medical students is influenced by various factors, including personal preferences, perceptions of the specialty, and educational experiences. Understanding these factors is crucial for addressing challenges and fostering interest in surgical specialties.

Methodology: This study analyzed data from 889 medical students to explore demographic characteristics and factors influencing their decision regarding pursuing a surgical career. Participants completed surveys regarding their gender, academic year, GPA, and intentions regarding surgical careers. Factors influencing career were assessed using closed-ended questions, with both deterrents and motivators considered.

Results: The study found a higher proportion of female participants (63.1%) compared to male participants (36.9%). Factors influencing participants' decision not to pursue a surgical career included a preference for other specialties (61.8%), a dislike for the lifestyle of surgeons (51.3%), and perceptions of long training durations (33.7%). Conversely, interest in the specialty (71.0%) and a preference for hands-on work (55.6%) were significant motivators for pursuing a surgical career. Additional factors such as concerns about the competitive environment, dislike for hands-on work, and perceptions regarding salary and work environment also emerged as notable considerations.

Conclusion: The findings highlight the importance of addressing misconceptions, providing mentorship, and promoting diversity within surgical specialties to encourage interest and diversify the surgical workforce. The study found a high interest in surgical specialty among Saudi medical students; however, some barriers were identified, particularly work-home relations, gender disparity, and pressure put on surgeries.

## Introduction

There are many great factors that affect medical students’ decisions when it comes to choosing a specialty, as it can be a very complex and critical process [1]. Lifestyle considerations, personality fit, income potential, and the presence of a role model in the interesting field are all aspects that are believed to influence medical students’ choice of specialty [2]. Moreover, the multifactorial impact has affected the students’ career choices and caused a decline in the surgical field in both interest and recruitment among students and led to a shortage in the specialty [3]. Former researchers have found that the reason for this shortage can be due to students deciding on their interest in the study when they perform the clinical examination and the bedside teaching, and due to the lack of engagement with surgery in medical school, it has led to the decline in the specialty along with the others, such as the undesirable lifestyle of surgeons and burnout associated with surgical training [4,5]. Gender inequalities have been a major issue, where many studies suggested that the possible explanation for this was motherhood, as many surgical residency programs lack a formal institutional maternity leave policy, contributing to the uncertainty for female medical students of how they will be able to balance their family and surgical training. However, in this day and age, both males and females value careers that allow them to have a life outside of work and have more time for social activities [6]. When it comes to improving and expanding the surgical specialty, studies have shown that increasing medical students’ exposure through positive mentorship and activities that mimic surgical procedures can lead to an effective outcome [7]. Our purpose in this study is to identify the factors that influence medical students in Saudi Arabia when it comes to choosing a surgical specialty and to understand the discouraging factors that lead to the decline in the surgical specialty.

Research objectives

The aim of the current study was to determine the prevalence of interest in surgical specialties among medical students in Saudi Arabia. In addition, we aimed to identify and compare factors that motivate and deter medical students in Saudi Arabia from pursuing surgical careers and to investigate the relationship between prior operating room exposure and interest in surgery among medical students in Saudi Arabia.

## Materials and methods

The methodology employed for this research study involved a cross-sectional approach utilizing a questionnaire-based survey conducted via social media platforms including WhatsApp, Twitter, and Telegram. The study aimed to investigate non-medical factors influencing the motivation of medical students from different universities across the Kingdom of Saudi Arabia to pursue a career in surgery.

Participants in the study consisted of medical students enrolled in both public and private medical colleges across Saudi Arabia, spanning from the first year to the sixth year of study. The sample population was determined to be approximately 377 students, with a 95% confidence level and a 5% margin of error, as estimated through Raosoft.com. Inclusion criteria comprised medical students and medical interns studying within Saudi Arabia, while exclusion criteria excluded practicing doctors and Saudi students pursuing medical education outside the country. Recruitment of participants was conducted through random sampling techniques. Invitations to participate in the study were disseminated via Google Forms, ensuring equal opportunity for all students to respond. An informed consent statement was presented to participants at the outset of the questionnaire, guaranteeing confidentiality and privacy of their responses.

The primary task assigned to participants was the completion of an anonymous, structured online questionnaire designed using Google Forms, where links to the questionnaire were distributed throughout different medical groups and pages in social media. The questionnaire included questions regarding participants' interest in surgery and their perceptions of the standard of surgical training within Saudi Arabia. The questionnaire was self-prepared by the authors depending on the literature review [8-9]. It was tested among a small sample size of 10 participants to assess its validity and readability. There is no need for any change in the main questionnaire after the pilot study. Additionally, participants were asked closed-ended multiple-choice questions regarding prior exposure to surgery, specific surgical specialty interests, and perceived motivators and barriers to pursuing a surgical career. In addition, the questionnaire was started with a consent agreement to participate in the study. Those who refused to participate were excluded from the study. Data collected from the questionnaire were stored and managed using Microsoft Excel (Microsoft Corporation, Redmond, USA) to maintain confidentiality. Participants with missing responses or incomplete responses were excluded from the study.

The expected outcomes of the study aimed to identify factors influencing medical students' decisions regarding pursuing a career in surgery. Given the anticipated decline in interest in surgical specialties among medical students nationally and internationally, this research sought to shed light on pertinent issues that could potentially address misconceptions and motivate students to consider surgery as a career path.

Following data collection, analysis was conducted using IBM SPSS Statistics for Windows, Version 28 (Released 2021; IBM Corp., Armonk, New York, USA). Frequency and percent were used for the description of categorical variables. Independent samples T-test, chi test, and ANOVA were used to determine the factors affecting students' decisions. All statements were considered significant when the p-value was lower than 0.05. This facilitated comprehensive statistical analysis to identify trends, correlations, and associations among variables investigated in the study.

The study was conducted after getting approval from the Institutional Review Board of Imam Mohammad Ibn Saud Islamic University (approval no. 623/2024). No personal data were collected from the participants, and all data was saved on the laptop of the main authors and saved in a protected file with a password.

The dissemination of findings from this research served to address potential challenges within the surgical field by implementing programs aimed at increasing student exposure to surgical specialties from the early years of medical education and fostering mentorship relationships. By sharing the final findings, opportunities were created to enhance understanding and interest in surgical careers among medical students in Saudi Arabia.

## Results

The analysis of Table 1 reveals the demographic distribution of participants in the study. Among the 889 respondents, 36.9% were male (N=328) and 63.1% were female (N=561). Concerning the medical colleges represented, the highest proportion of participants hailed from the Al-Imam Muhammad bin Saud Islamic Medical College (174, 19.6%), followed by King Faisal Medical College (94, 10.6%), and Private Medical College (82, 9.2%). In terms of academic year, the majority of participants were in their second year (197, 22.2%), followed by third year (188, 21.1%), and fifth year (151, 17%). Notably, most participants reported their GPA on a scale out of 5 (588, 66.1%), with the majority falling within the range of 4.76-5.00 (272, 30.6%).

**Table 1:** Demographic factors of the participants

		Count	Column N %
Gender	Male	328	36.9%
	Female	561	63.1%
College	Al-Imam Muhammad bin Saud Islamic Medical College	174	19.60%
	King Faisal Medical College	94	10.60%
	Private Medical College	82	9.20%
	King Saud Bin Abdulaziz Universities for Health Sciences - Medical College	77	8.70%
	Taibah Medical College	75	8.40%
	Al Jouf Medical College	51	5.70%
	King Saud Medical College	50	5.60%
	Taif Medical College	49	5.50%
	Jazan Medical College	42	4.70%
	Northern Border Medical College	41	4.60%
	Princess Nourah Bint Abdulrahman Medical College	28	3.10%
	King Abdulaziz Medical College	25	2.80%
	Ummul Qura Medical College	24	2.70%
	Majmaah Medical College	17	1.90%
	Imam Mohammed Bin Faisal Medical College	14	1.60%
	Jeddah Medical College	11	1.20%
	Qassim Medical College	10	1.10%
	Al Baha Medical College	7	0.80%
	Tabuk Medical College	6	0.70%
	Prince Sattam Bin Abdulaziz Medical College	5	0.60%
	King Khalid Medical College	3	0.30%
	Shagra Medical College	3	0.30%
	Najran Medical College	1	0.10%
Academic year	Pre-medical (preparatory year)	65	7.3%
	1st year	93	10.5%
	2nd year	197	22.2%
	3rd year	188	21.1%
	4th year	145	16.3%
	5th year	151	17.0%
	Internship	50	5.6%
GPA (out of 5)	My GPA is out of 4	162	18.2%
	2.01-2.5	12	1.3%
	2.51-3.00	16	1.8%
	3.01-3.50	33	3.7%
	3.51-4.00	76	8.5%
	4.01-4.50	147	16.5%
	4.51-5.75	171	19.2%
	4.76-5.00	272	30.6%
GPA (out of 4)	My GPA is out of 5	588	66.1%
	2.01-2.5	16	1.8%
	2.51-3.00	32	3.6%
	3.01-3.50	84	9.4%
	3.51-4.00	169	19.0%
Have you ever thought to pursue a career in the surgical field?	No	199	22.4%
	Yes	483	54.3%
	Have not decided yet	207	23.3%
The data has been represented as N, %			

**Figure 1:**
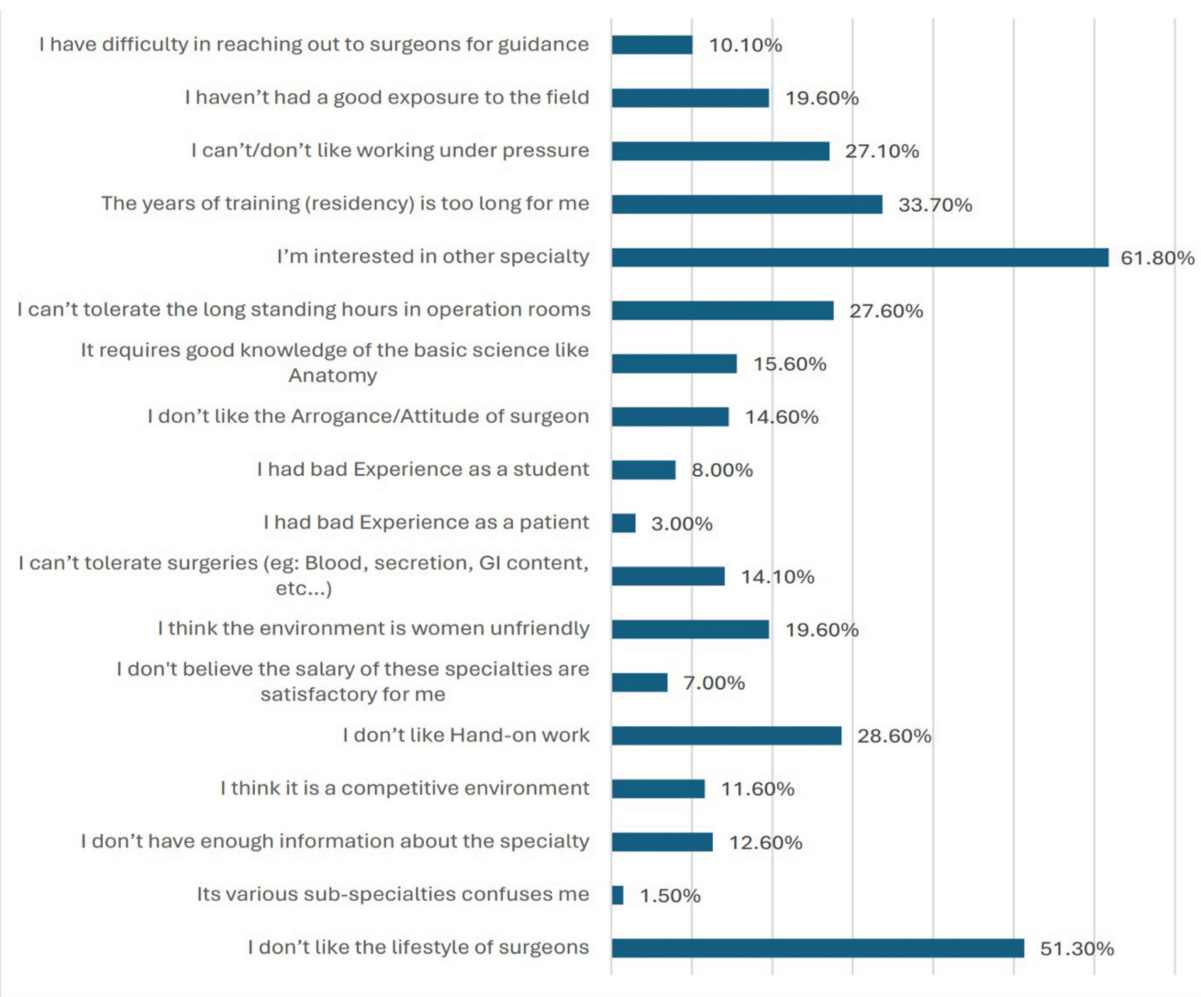
Factors influencing participants’ decision not to pursue a career in the surgical field

Figure 1 illustrates the factors influencing participants' decision not to pursue a career in the surgical field. The most prominent deterrents included a preference for other specialties (549, 61.8%), a dislike for the lifestyle of surgeons (456, 51.3%), and the perception of long training durations (299, 33.7%). Besides the prominent deterrents mentioned earlier, other notable factors influencing the decision not to pursue a surgical career include concerns about the competitive environment (103, 11.6%), dislike for hands-on work (254, 28.6%), and apprehension toward surgical procedures due to factors such as blood, secretions, or gastrointestinal content (125, 14.1%). Additionally, perceptions regarding the salary of surgical specialties being unsatisfactory (62, 7.0%) and concerns about the environment being unfriendly toward women (174, 19.6%) also emerged as significant considerations.

**Figure 2:**
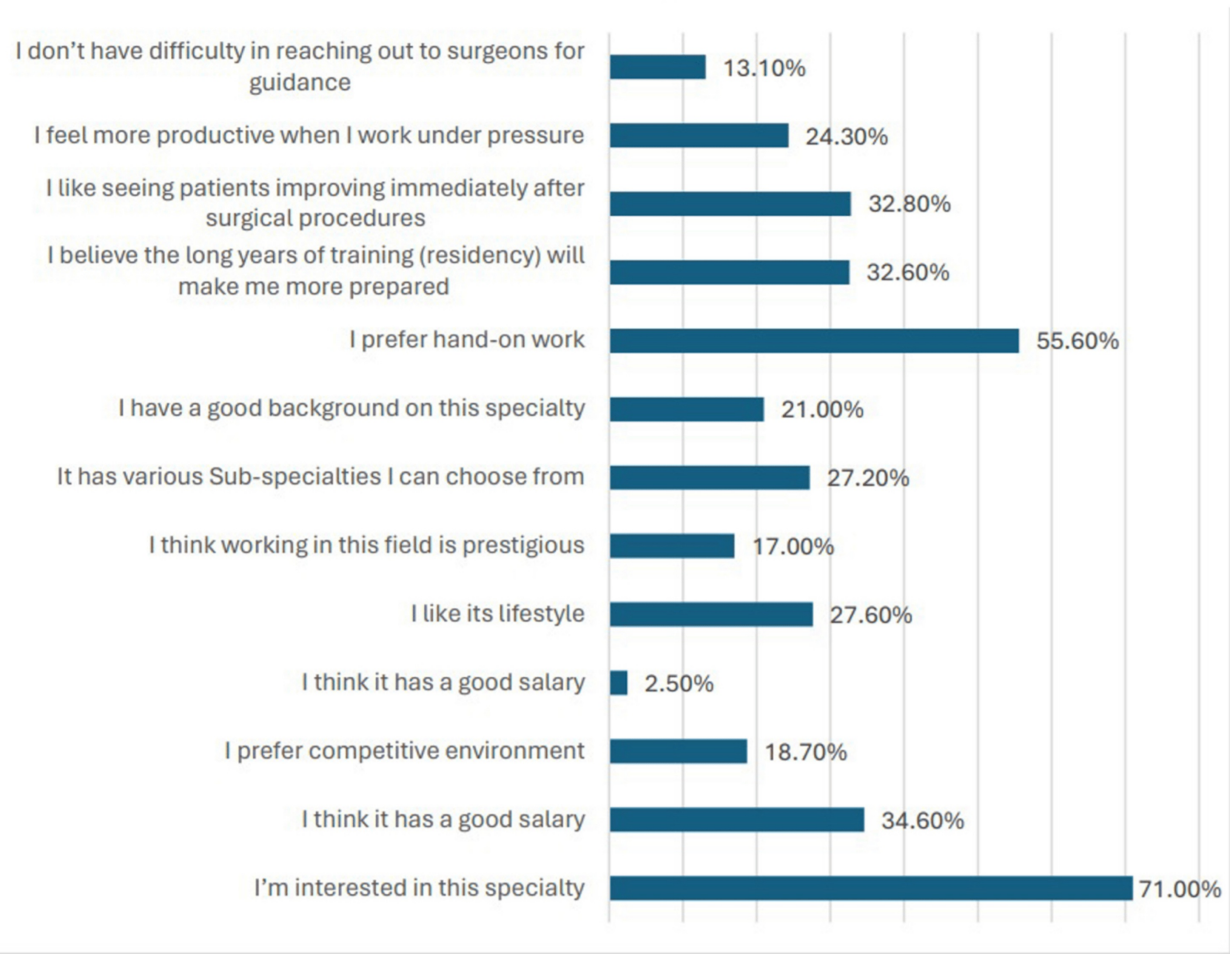
Factors influencing participants’ decision in pursuing a career in the surgical field

Conversely, Figure 2 highlights the factors that contribute to participants' inclination toward a surgical career, with interest in the specialty (631, 71.0%) and a preference for hands-on work (494, 55.6%) being the most significant motivators. Beyond the highlighted motivators, other factors contributing to participants' inclination toward a surgical career include the perception of surgery as a prestigious field (151, 17.0%), the appeal of various sub-specialties within surgery (241, 27.2%), and the belief that long years of training (residency) will enhance preparedness (290, 32.6%). Moreover, the desire to witness immediate patient improvement post-surgical procedures (292, 32.8%) and the feeling of increased productivity when working under pressure (216, 24.3%) were also notable factors driving interest in surgical specialties among participants.

However, Table 2 delves into the characteristics associated with an interest in the surgical field among participants. Among those who had not yet decided on pursuing a surgical career, factors such as lack of exposure to the field (30.6%) and uncertainty regarding long-term lifestyle preferences (20.9%) were prevalent. Additionally, the majority of participants who expressed interest in surgery developed this inclination during their pre-medical or preparatory year (33.6%).

**Table 2:** Characteristics of being interested in the surgical field GYN: gynecology

		Count	Column N %
If you answered 'I have not decided yet' what are the factors that affected your decision?	I still have not had any exposure to the field.	60	30.6%
	I am not sure if I prefer medical or surgical specialties.	37	18.9%
	I am not sure about pursuing the lifestyle long-term.	41	20.9%
	There are too many sub-specialties I want to explore.	48	24.5%
	I am not sure about the future of surgical specialties.	10	5.1%
Since when did you develop interest in the surgical field?	Pre-Medical (Preparatory Year)	299	33.6%
	1st year	74	8.3%
	2nd year	57	6.4%
	3rd year	81	9.1%
	4th year	65	7.3%
	5th year	36	4.0%
	Internship	12	1.3%
	I am not interested	265	29.8%
If you are interested in pursuing a surgical career, which specialty have you decided on?	I have no interest in surgery	227	25.5%
	General	86	9.7%
	Neurosurgery	95	10.7%
	ENT	42	4.7%
	Cardiac surgery	75	8.4%
	Plastic	39	4.4%
	Orthopedic	47	5.3%
	Ophthalmology	39	4.4%
	GYN	28	3.1%
	Pediatric	39	4.4%
	Urology	8	0.9%
	Undecided	164	18.4%
The most significant barrier to pursue a career in surgery?	I do not like the lifestyle of the surgeons	242	27.2%
	It requires good knowledge of the basic sciences like anatomy.	48	5.4%
	I have difficulty in reaching out to surgeons for guidance	29	3.3%
	I do not prefer working under pressure	84	9.4%
	I cannot tolerate the long-standing hours in operating rooms.	79	8.9%
	I did not have enough exposure to surgical specialties (Elective curriculum in college)	84	9.4%
	The competitive environment is unattractive to me.	57	6.4%
	l have more interest in pursuing an academic career	85	9.6%
	I like to see and follow the patient for a long period of time	55	6.2%
	Their salary is not enough compared to the workload.	48	5.4%
	I do not prefer that it is mostly hands on work.	50	5.6%
	It has various sub-specialty, which confuses me	28	3.1%
Do you have any prior exposure to operating room?	No	581	65.4%
	Yes	308	34.6%
IF YES: Time of first contact with the operating room	1st year	34	11.0%
	2nd year	33	10.7%
	3rd year	73	23.7%
	4th year	128	41.6%
	5th year	29	9.4%
	Internship	11	3.6%
IF YES: Did this experience affect your decision?	Yes, the experience played a role in my interest in surgical specialties	162	52.6%
	Yes, the experience played a role in my lack of interest in surgical specialties	65	21.1%
	No, it had no influence on my decision	81	26.3%
The data has been represented as N, %			

The relationship between demographic factors and the intention to pursue a surgical career is explored in Table 3. Gender appeared to influence career aspirations, with a higher proportion of males expressing intent to pursue surgery compared to females (204, 62.2% vs. 279, 49.7%). Similarly, the academic year exhibited a significant association, with a greater proportion of students in their third and fourth years expressing interest in surgery compared to those in other years (89, 47.3% and 80, 55.2%, respectively).

**Table 3:** The relation between demographic factors and the intention to have a career in the surgical field

		Have you ever thought to pursue a career in the surgical field?						
		No		Yes		Have not decided yet		
		Count	Row N %	Count	Row N %	Count	Row N %	
Gender	Male	57	17.4%	204	62.2%	67	20.4%	0.001^*^
	Female	142	25.3%	279	49.7%	140	25.0%	
Academic year	Pre-medical (preparatory year)	3	4.6%	45	69.2%	17	26.2%	0.002^*^
	1st year	16	17.2%	50	53.8%	27	29.0%	
	2nd year	38	19.3%	110	55.8%	49	24.9%	
	3rd year	47	25.0%	89	47.3%	52	27.7%	
	4th year	35	24.1%	80	55.2%	30	20.7%	
	5th year	42	27.8%	83	55.0%	26	17.2%	
	Internship	18	36.0%	26	52.0%	6	12.0%	
GPA (out of 5)	My GPA is out of 4	36	22.2%	89	54.9%	37	22.8%	0.363
	2.01-2.5	0	0.0%	8	66.7%	4	33.3%	
	2.51-3.00	4	25.0%	7	43.8%	5	31.3%	
	3.01-3.50	5	15.2%	18	54.5%	10	30.3%	
	3.51-4.00	20	26.3%	36	47.4%	20	26.3%	
	4.01-4.50	37	25.2%	86	58.5%	24	16.3%	
	4.51-5.75	44	25.7%	83	48.5%	44	25.7%	
	4.76-5.00	53	19.5%	156	57.4%	63	23.2%	
GPA (out of 4)	My GPA is out of 5	139	23.6%	305	51.9%	144	24.5%	0.058
	2.01-2.5	3	18.8%	6	37.5%	7	43.8%	
	2.51-3.00	9	28.1%	21	65.6%	2	6.3%	
	3.01-3.50	17	20.2%	53	63.1%	14	16.7%	
	3.51-4.00	31	18.3%	98	58.0%	40	23.7%	
The data has been represented as N, %. ^*^Significant at p-value<0.05								

## Discussion

Surgery stands as a cornerstone in the medical field, offering crucial interventions that can alleviate suffering, extend lives, and enhance quality of life. It is a dynamic and challenging specialty that requires a blend of technical skill, critical decision-making, and compassion. The high tendency of medical students to desire a career in surgery underscores its significance within the healthcare landscape. This study aims to delve into the factors influencing medical students in Saudi Arabia when considering a surgical specialty, as well as to comprehend the deterrents leading to a decline in interest in this field.

The prevalence of individuals who have contemplated pursuing a career in the surgical field, as indicated by the findings (54.3%), reflects a substantial portion of the medical student population, especially those interested in neurosurgery and cardiac surgery. This prevalence aligns with global trends where surgery continues to attract a significant proportion of aspiring physicians. A previous study conducted at Jazan University, Saudi Arabia, reported that out of 413 participants, 74.3% were considering a surgical career, with 24.4% interested in general surgery, followed by cardiac surgery (14.3%) and pediatric surgery (12.4%) [8]. However, another study in Canada showed that only 20 (18.7%) and 30 (28.0%) out of 107 medical students listed surgical and non-surgical disciplines as their current career interests [9], and another study in China showed that 63.76% expressed a willingness for a surgical career, with “interest” being a key driving factor (73.41%) [10]. In addition, our results are higher than reported in two different studies conducted at Jena, in Germany, and among Mexican medical students reported a prevalence of students interested in surgery of 25% and 52.4% [11,12].

However, it is essential to dissect this prevalence further to understand its nuances. Gender and academic year emerge as crucial demographic factors shaping career aspirations. Males exhibit a higher inclination toward surgery compared to females, which resonated with existing literature highlighting a gender disparity in surgical specialties and showed that female medical students are significantly less likely to choose a surgical specialty [10,13-16]. One possible explanation for these results, reported by a previous study, showed that females were more likely to agree that their chance of becoming a spouse could be affected by a surgical career (p=0.002) and that meeting role models could influence their choice of surgical specialty (p=0.015) [8].

Another possible reason for the low tendency of females to choose the surgical field is gender discrimination and bias in surgical specialties, which have been reported in multiple studies. For instance, a study conducted in 2020 found that female surgeons experienced prejudice in various forms, including differential treatment and encountering comments related to their gender [17]. Additionally, research has revealed that female surgeons face obstacles such as a lack of mentorship, income disparities, and concerns regarding work-life balance [18]. Sexual harassment and discrimination have emerged as significant barriers during surgical residency and fellowship training [19]. Furthermore, a survey conducted in 2020 indicated that female medical students are significantly less inclined to pursue a surgical specialty, potentially due to perceived constraints such as long workdays, limited leisure time, and societal or cultural barriers [20]. Despite advancements in addressing gender disparity in surgery, there remains a pressing need to ensure that women have equal opportunities to pursue and thrive in surgical careers [21]. Similarly, the academic year showcases a notable association, with a higher proportion of students in their third- and fourth years expressing interest in surgery. This trend may reflect the progressive exposure to clinical environments and specialty rotations, which often solidify career preferences among medical students. Both age and decreased interest in research were associated with medical specialty interest, which is reflected in other studies [22,23]. Interestingly, student interest in academic medicine has historically been associated with older age [24].

Examining the reasons against choosing a surgical field as a specialty unveils a spectrum of deterrents. Preferences for other specialties, concerns about the lifestyle of surgeons, and perceptions of long training durations feature prominently among these deterrents. Such findings resonate with the broader discourse surrounding surgical careers, where lifestyle considerations, including long and demanding work hours, remain significant deterrents, particularly among younger generations of physicians [25-27]. Additionally, concerns about the competitive environment, aversion to hands-on work, and apprehensions toward surgical procedures further contribute to the reluctance to pursue a career in surgery.

Conversely, exploring the motivators for choosing a surgical specialty sheds light on the factors driving interest among medical students. Intriguingly, interest in the specialty itself and a preference for hands-on work emerge as predominant motivators. This underscores the allure of surgery as a dynamic and impactful field that offers direct patient care and tangible outcomes. Peel and colleagues found that students interested in surgery tend to consider lifestyle as less important when making career decisions, while students not interested in surgery highly valued lifestyle [28]. Moreover, the perceived prestige of surgery, the appeal of various sub-specialties within the field, and the belief in the value of extensive training further bolster interest among aspiring surgeons [29,30]. The desire to witness immediate patient improvement post-surgical procedures and the inclination toward high-pressure environments highlight the intrinsic rewards associated with surgical practice.

Identifying the most significant barrier to pursuing a career in surgery reveals multifaceted challenges. The dislike for the lifestyle of surgeons stands out prominently as a deterrent, emphasizing the importance of work-life balance and well-being considerations in career decision-making [22,24]. Additionally, concerns about long-standing hours in operation rooms, competitive environments, and the perceived inadequacy of salaries relative to workload underscore systemic issues within surgical practice that need addressing to foster a more inclusive and sustainable workforce [26].

Last, evaluating prior exposure to the operating room unveils its pivotal role in shaping career decisions. A substantial proportion of participants lacked exposure to the operating room, highlighting the need for enhanced clinical experiences and mentorship opportunities to foster interest in surgery. Interestingly, for those with prior exposure, the experience significantly influenced their career decisions, whether positively or negatively. This underscores the transformative impact of hands-on experiences in shaping career trajectories and emphasizes the importance of early exposure to surgical specialties during medical education.

Limitations

Despite the valuable insights gained from this study, several limitations must be acknowledged. First, the use of a cross-sectional design limits the ability to infer causality between the identified factors and the decision to pursue a surgical career. Additionally, the reliance on self-reported data may introduce response bias, as participants might overestimate or underestimate their motivations and deterrents. The recruitment of participants via social media platforms could also result in a selection bias, potentially excluding students who are less active on these platforms. Furthermore, the study's focus on medical students within Saudi Arabia may limit the generalizability of the findings to other regions or countries with different educational systems and cultural contexts. Last, the questionnaire's structured format may restrict the depth of responses, potentially overlooking nuanced factors influencing career decisions. Future research employing longitudinal designs, broader sampling methods, and qualitative approaches could provide a more comprehensive understanding of the factors shaping medical students' career aspirations in surgery.

## Conclusions

In conclusion, this study provides valuable insights into the factors influencing medical students' decision regarding pursuing a surgical career in Saudi Arabia. The study showed a good percentage of students who intended to contemplate pursuing a career in the surgical field, with higher intention reported among male students. Preferences for other specialties, concerns about the lifestyle of surgeons, and perceptions of long training durations feature prominently among reasons against choosing a surgical field, while intriguingly, interest in the specialty itself and a preference for hands-on work emerge as predominant motivators. By elucidating both the deterrents and motivators shaping career aspirations, this research contributes to a deeper understanding of the dynamics within surgical specialties. Addressing the identified barriers while bolstering opportunities for exposure and mentorship holds promise in fostering a diverse and resilient surgical workforce capable of meeting the evolving healthcare needs of society.

## Appendices

The questionnaire

Section A: General questions

1-  Gender:

●      Male

●      Female

2-  College:

●      Al-Imam Muhammad bin Saud Islamic Medical College

●      Princess Nourah Bint Abdulrahman Medical College

●      Majmaah Medical College

●      King Faisal Medical College

●      Qassim Medical College

●      Imam Mohammed Bin Faisal Medical College

●      Prince Sattam Bin Abdulaziz Medical College

●      King Abdulaziz Medical College

●      King Khalid Medical College

●      King Saud Bin Abdulaziz Universities for Health Sciences - Medical College

●      King Saud Medical College

●      Shagra Medical College

●      Taibah Medical College

●      Northern Border Medical College

●      Tabuk Medical College

●      Najran Medical College

●      Jazan Medical College

●      Al Jouf Medical College

●      Al Baha Medical College

●      Jeddah Medical College

●      Ummul Qura Medical College

●      Taif Medical College

●      Private Medical College

3-  Academic year:

●      Pre-medical (preparatory year)

●      1st year

●      2nd year

●      3rd year

●      4th year

●      5th year

●      Internship

4-  GPA (out of 5):

●      4.76-5.00

●      4.51-4.75

●      4.01-4.50

●      3.51-4.00

●      3.01-3.50

●      2.51-3.00

●      2.01-2.50

●      My GPA is out of 4

5-  GPA (out of 4):

●      3.51-4.00

●      3.01-3.50

●      2.51-3.00

●      2.01-2.50

●      My GPA is out of 5

Section B: Factors influencing medical students’ decision in choosing a surgical specialty.

6-  Have you ever thought to pursue a career in the surgical field?

●      Yes

●      No

●      Haven’t decided yet

7-  If you answered "NO" what are the factors that affected your decision?

●      I haven’t had a good exposure to the field

●      I’m interested in other specialty

●      I don’t have enough information about the specialty

●      The years of training (residency) is too long for me

●      I have difficulty in reaching out to surgeons for guidance

●      I don’t like hand-on work

●      I think the environment is women unfriendly.

●      I don’t like the lifestyle of surgeons

●      I don't believe the salary of these specialties are satisfactory for me.

●      I think it is a competitive environment.

●      I can’t tolerate surgeries (eg: Blood, secretion, GI content, etc…)

●      Its various sub-specialties confuses me

●      I don’t like the arrogance/attitude of surgeons

●      It requires good knowledge of basic science like Anatomy

●      I can’t tolerate the long standing hours in operation rooms

●      I can’t/don’t like working under pressure.

●      I had bad Experience as a student

●      I had bad Experience as a patient

●      I Answered "YES"

●      I Answered "I haven’t decided yet"

7-  If you answered "Yes" what are the factors that affected your decision?

●      I’m interested in this specialty

●      I have a good background on this specialty

●      I believe the long years of training (residency) will make me more prepared.

●      I don’t have difficulty in reaching out to surgeons for guidance

●      I prefer hand-on work

●      I like its lifestyle

●      I think it has a good salary

●      I prefer competitive environment

●      It has various sub-specialties I can choose from

●      I think working in this field is prestigious

●      I like seeing patients improving immediately after surgical procedures

●      I feel more productive when I work under pressure

●      I answered NO

●      I Answered "I haven’t decided yet"

8-  If you answered "I haven’t decided yet" what are the factors that affected your decision?

●      I am not sure about pursuing the lifestyle long-term

●      There are too many sub-specialties I want to explore

●      I am not sure about the future of surgical specialties

●      I still haven't had any exposure to the field

●      I am not sure if I prefer medical or surgical specialties

●      I answered “Yes”

●      I Answered “No”

9-Since when you developed the interest in the surgical field?

●      Before pre-medical (preparatory year)

●      pre-medical (preparatory year)

●      1st year

●      2nd year

●      3rd year

●      4th year

●      5th year

●      Internship

●      I’m not interested

10-  If you are interested in pursuing a surgical career, which specialty have you decided on?

●      Undecided

●      General

●      Cardiac surgery

●      Pediatric

●      Plastic

●      Neurosurgery

●      Orthopedic

●      Ophthalmology

●      ENT

●      Urology

●      GYN

●      I Have no interest in surgery

11- The most significant barrier to pursue a career in surgery?

●      l have more interest in pursuing an academic career

●      I don’t prefer that it's mostly hands on work

●      I like to see and follow the patient for a long period of time

●      I don’t like the lifestyle of the surgeons

●      I think their salary is not enough compared to the workload

●      I have difficulty in reaching out to surgeons for guidance

●      The competitive environment is unattractive to me

●      It has various sub-specialty, which confuses me

●      I did not have enough exposure to surgical specialties (Elective curriculum in college)

●      It requires good knowledge of basic science like anatomy

●      I don’t prefer working under pressure

●      I can’t tolerate the long standing hours in operation rooms

12-  Do you have any prior exposure to the operating room?

●      Yes

●      No

13-  IF YES: Time of first contact with the operating room

●      1st year

●      2nd year

●      3rd year

●      4th year

●      5th year

●      Internship

●      I answered No

14-  IF YES: Did this experience affect your decision?

●      Yes, the experience played a role in my interest in surgical specialties

●      Yes, the experience played a role in my lack of interest in surgical specialties

●      No, it had no influence on my decision

●      I answered No
